# Barriers to Ophthalmologic Care Reported by Family Caregivers of Users and Non‐Users With Intellectual Disabilities

**DOI:** 10.1111/jir.70123

**Published:** 2026-06-02

**Authors:** Chiun‐Ho Hou, Yueh‐Ching Chou, Shu‐Fang Shih, Christy Pu

**Affiliations:** ^1^ Department of Ophthalmology National Taiwan University Hospital Taipei Taiwan; ^2^ Department of Social Work Asia University Taipei Taiwan; ^3^ Department of Health Administration, College of Health Professions Virginia Commonwealth University Richmond Virginia USA; ^4^ Institute of Public Health National Yang Ming Chiao Tung University Taipei Taiwan

## Abstract

**Background:**

People with intellectual disabilities (IDs) are more likely to experience vision‐related impairments, yet they face many barriers to accessing eye care. Although previous studies have described these barriers, few have compared barriers reported by families who have and have not accessed ophthalmologic care, which may provide insight into barriers interpreted as anticipated or experienced.

**Objective:**

The study objectives are to compare barriers to ophthalmologic care reported by family caregivers of users and non‐users and to interpret these differences as anticipated or experienced barriers.

**Methods:**

A cross‐sectional survey was conducted with 657 primary family caregivers in Taichung City and Hualien County, Taiwan, between August and October 2023. Caregivers selected from a list of 13 predefined barriers. Participants were classified as ‘users’ (*n* = 261) if they had taken their family member with IDs for an eye examination and ‘non‐users’ (*n* = 396) otherwise. For interpretation, barriers reported significantly more often by non‐users were labelled as ‘anticipated’, whereas those reported significantly more often by users were labelled as ‘experienced’; these labels were based on service use status rather than direct temporal measurement. Logistic regression with marginal effects was used to identify predictors of the most common anticipated barrier.

**Results:**

A clinic environment perceived as unwelcoming or inaccessible was reported by 17.6% of non‐users and 4.5% of users (*p* < 0.001). Users more frequently reported difficulty participating during appointments or examinations (9.95% vs. 4.25%, *p* = 0.004), equipment that could not be adapted (8.0% vs. 4.3%, *p* = 0.043), limited availability of accessible assessment tools (7.1% vs. 3.5%, *p* = 0.043) and limited provider experience in delivering accessible care for people with IDs (7.6% vs. 2.6%, *p* = 0.003). Higher caregiver strain and having ≥ 2 vision‐related conditions were associated with reporting the environmental barrier.

**Conclusion:**

Barriers reported by non‐users suggest that perceptions of clinics as unwelcoming or inaccessible may discourage families from seeking a first eye examination for people with IDs. Improving accessibility and the care environment, alongside staff training and adaptable equipment and assessment tools, may reduce barriers reported by both non‐users and users.

## 1 Introduction

Vision plays an important role in everyday life for people with intellectual disabilities (IDs) (Evenhuis et al. 2009). Studies across multiple countries have consistently found that people with IDs are at substantially higher risk of having undetected or untreated vision‐related impairments than non‐disabled people (Evenhuis et al. 2001; Van Splunder et al. 2006; Kroezen et al. 2020). Despite this elevated risk, people with IDs and their families continue to face persistent and multifaceted barriers to accessing ophthalmologic care (Chou et al. 2025; Li et al. 2015).

Previous research has identified a range of obstacles to eye care for people with IDs, including limited awareness of available services, physically inaccessible clinic environments, lack of adapted assessment instruments, communication barriers and limited provider training in delivering accessible care (Li et al. 2015; Ali et al. 2013). Much of this literature draws on the perspectives of caregivers and people with IDs themselves (Gibbs et al. 2008; McCormick et al. 2021; Iacono et al. 2014; Webber et al. 2010). However, some barriers may be anticipated before any appointment is sought, shaping expectations and discouraging initial service use (Donnelly et al. 2017). Understanding whether barriers are anticipated in advance or encountered during care matters for intervention design, because barriers that deter first contact may require different strategies than those that arise within clinical encounters.

Addressing barriers that prevent families from scheduling an initial appointment requires targeted outreach and clear information about accessible services. For example, appropriately designed clinical settings can support effective eye care for people with IDs (Li et al. 2015). Yet, caregivers may assume that such settings, supports or accommodations are unavailable, which can deter them from seeking care. By contrast, barriers that emerge during care—such as difficulties participating in examination procedures, equipment that cannot be adapted or communication that does not meet the person's needs—are more likely to become apparent once services are accessed. Distinguishing between *anticipated* and *experienced* barriers may therefore enable more strategic responses: Community‐facing information and environmental improvements can support initial contact, whereas staff training and service redesign can improve the accessibility and quality of care during appointments.

A useful conceptual framework for understanding barriers to care is the patient‐centred access to healthcare model proposed by Levesque et al. (2013). This model conceptualises access as a dynamic interface between supply‐side dimensions of the health system (approachability, acceptability, availability and accommodation, affordability and appropriateness) and demand‐side abilities of patients to perceive, seek, reach, pay for and engage in care. It highlights that barriers can arise both before care is sought and after services are reached and used. This perspective is particularly relevant for examining how caregivers of people with IDs describe barriers in relation to whether ophthalmologic services have ever been used.

In this study, we draw on this framework to interpret differences in barriers reported by caregivers of ‘users’ and ‘non‐users’ of ophthalmologic services. The study objectives are (1) to identify and quantify barriers to ophthalmologic care for people with IDs, (2) to compare barriers reported by users and non‐users and interpret these differences as anticipated or experienced barriers and (3) to examine whether service use is associated with caregivers' perceptions of vision‐related conditions among people with IDs. We hypothesise that people with IDs encounter multiple barriers to ophthalmologic care (Objective 1). We also hypothesise that the types of barriers differ between those who have accessed ophthalmologic care and those who have not, specifically, that barriers reported more often by non‐users may reflect anticipated difficulties related to seeking care, whereas barriers reported more often by users may reflect challenges encountered during care (Objective 2). Because identifying vision‐related conditions often depends on professional assessment, we also hypothesise that caregivers in the user group will report a higher number of such conditions than caregivers in the non‐user group (Objective 3).

## 2 Methods

### 2.1 Research Design and Participants

This study conducted a cross‐sectional survey in Taiwan. Data were collected between 15 August and 5 October 2023 through in‐person interviews. Trained interviewers visited the homes of eligible participants to conduct face‐to‐face interviews. The target population was primary family caregivers of individuals with IDs who were officially registered in the Disability Registries of Taichung City and Hualien County. Eligible individuals were those who were caregivers of people with IDs that had been formally assessed and classified by local authorities and who were under the age of 65 years as of July 2023.

To be included in the study, individuals were required to meet the following criteria: (1) being the primary family caregiver of a family member with IDs aged between 0 and 64 years and (2) being between 20 and 99 years of age. Individuals who were unable to communicate in Mandarin were excluded from participation.

Throughout the entire survey, for questions related to the person with ID's own perceptions, caregivers were instructed to confirm their answers with that person whenever possible.

### 2.2 Ethics Approval and Consent to Participate

This study was approved by the Institutional Review Board of Chang Gung Memorial Hospital, Taiwan (approval number: 202200078B0). Informed consent was obtained from each participant.

### 2.3 Sampling Design and Weighting

The sampling frame for this study was derived from the disability registries of two administrative regions in Taiwan, namely, Taichung City and Hualien County. These locations were deliberately selected to reflect Taiwan's geographic and socio‐economic diversity. Taichung is a more urbanised and economically developed area, whereas Hualien is rural and less affluent. Including both regions enabled us to capture a broad range of lived experiences within the disability community, which enhanced the representativeness and relevance of the findings for national‐level policy and service planning. Details of the sampling design were previously published elsewhere (Hou et al. 2025).

The registries used as the sampling frame include all residents with officially recognised disabilities in each jurisdiction. A stratified random sampling approach was used to ensure proportional representation, with stratification based on the distribution of people with IDs within each registry. According to registry data, Taichung City had 8787 registered individuals with IDs, and Hualien County had 1666.

Because of budgetary constraints, the target sample size was set at 650 participants. To ensure the study reflected regional population differences and maintained a minimum analytic sample from each area, 500 individuals were recruited from Taichung City and 150 from Hualien County. Participants were randomly selected from each registry by using computer‐generated random numbers. This sampling strategy ensured balanced representation of urban and rural populations and enabled regionally sensitive comparisons in the analysis.

### 2.4 Survey Instrument

This study is part of a broader public initiative aimed at improving access to vision care for people with IDs. The overarching objectives of the project include developing actionable strategies for vision care delivery, designing training modules for healthcare professionals and producing educational materials tailored to the needs of the caregivers of people with IDs. A qualitative study was first conducted to identify unmet needs and barriers related to ophthalmologic care in the target population.

The qualitative phase involved in‐depth interviews with 65 stakeholders involved in the vision care experience of people with IDs. The interview participants included (1) 20 individuals with IDs (18 with mild and 2 with moderate impairments); (2) 10 family caregivers; (3) 7 direct support or front‐line care workers; (4) 6 administrators or supervisors from healthcare and long‐term residential care settings; (5) 13 healthcare professionals, including optometrists, occupational therapists, ophthalmologists, family physicians, dentists and social workers; and (6) 9 representatives from non‐governmental organisations serving people with IDs. Additional details regarding this survey were provided in our previous publication (Hou et al. 2025).

On the basis of insights from the qualitative phase, we developed a structured questionnaire targeting primary family caregivers of people with IDs. The questionnaire included a list of 13 potential barriers to accessing ophthalmologic care derived from prior qualitative research and expert consultation. Caregivers were allowed to select multiple barriers they believed were applicable to them on the basis of their experience or concerns. The listed barriers were as follows:

1. The clinic environment is not friendly to people with IDs (e.g., limited consultation space, lack of disability‐friendly features, absence of fall prevention materials or a generally unwelcoming atmosphere) (environmental).
2. Health appointments or examinations may require communication support and accommodations (interpersonal).
3. Medical instruments and equipment are unsuitable for the persons with IDs (e.g., lack of portable devices for refraction or intraocular pressure measurement) (environmental).
4. There is a lack of assessment tools adapted for people with IDs (e.g., visual acuity charts and colour blindness tests) (environmental).
5. Healthcare personnel lack knowledge and skills related to IDs (e.g., difficulties in communicating with individuals with IDs) (systemic).
6. Healthcare personnel are impatient or unfriendly towards the person with IDs (interpersonal).
7. Other patients or their family members in the clinic are unfriendly (interpersonal).
8. The medical facility is too far away or transportation is inconvenient (environmental).
9. There is no time to take the person with IDs to the appointment (systemic).
10. The financial burden is too high, or treatment is too expensive (systemic).
11. I do not understand the different treatment options, making decision‐making difficult (systemic).
12. I am unable to secure an appointment with the desired physician (systemic).
13. I believe that the treatment would be ineffective (perceptual/systemic).

These response options were designed to capture both logistical and perceptual barriers from the caregiver's perspective, reflecting a broad spectrum of environmental, interpersonal and systemic challenges that affect access to vision care for people with IDs. Two items addressed the technical adaptation of ophthalmologic examinations for people with IDs. ‘Medical instruments and equipment are unsuitable for the person with intellectual disabilities’ referred primarily to physical devices used during eye examinations (e.g., slit lamps, autorefractors or tonometers). ‘There is a lack of assessment tools adapted for people with intellectual disabilities’ referred to the visual stimuli and testing materials used to evaluate vision (e.g., visual acuity charts and colour‐vision tests). Although this distinction may not be salient to all caregivers, we retained both items because they correspond to distinct practical targets for adapting ophthalmologic services (examination hardware vs. testing materials and procedures).

The questionnaire also included an item asking caregivers how often they brought the persons with IDs to see an ophthalmologist. Respondents could choose from four mutually exclusive options: “regularly”, “irregularly”, “not sure” and “never”. For analytical purposes, responses were dichotomised: “regularly” and “irregularly” were grouped as “users”, whereas “not sure” and “never” were categorised as “non‐users”.

### 2.5 Other Covariates

#### 2.5.1 Characteristics of the Persons With IDs

We included the age (in years at the time of survey) and sex (male/female) of the person with IDs. Functional status was assessed during the interview using overall scores from the Barthel Index for Activities of Daily Living (ADL) (Mahoney and Barthel 1965) and the Lawton and Brody Instrumental Activities of Daily Living (IADL) Index (Lawton and Brody 1969). ADL scores ranged from 0 to 100, and IADL scores ranged from 0 to 24, with higher scores indicating greater functional ability (Chou et al. 2007). Both instruments demonstrated strong internal consistency, with reliability coefficients of 0.92 for the ADL scale and 0.87 for the IADL scale (Chou et al. 2007). Individuals with ADL scores between 81 and 100 and IADL scores above 14 were classified as having mild impairment. Those with ADL scores between 61 and 80 and IADL scores between 0 and 14 were considered to have moderate impairment. Severe impairment was defined as ADL scores below 60 and IADL scores below 9 (Taipei City Government 2024). Finally, we asked whether the persons with IDs had private health insurance (yes/no).

#### 2.5.2 Characteristics of the Caregiver

Caregiver age (in years at the time of survey) and sex (male/female) were collected, along with marital status (married/other) and relationship with the persons with IDs (parent or parent‐in‐law/other). To determine socio‐economic status, we first asked caregivers to report their annual household income in NT$ brackets ranging from NT$200000 to NT$2400000. Because the responses at the upper end were sparse, income was collapsed into five categories: < NT$200 000; NT$200 000–NT$399 999; NT$400 000–NT$599 999; NT$600 000–NT$799 999; and ≥ NT$800 000. Because income alone may not fully reflect economic well‐being, we also assessed subjective financial satisfaction on a 5‐point scale (*very dissatisfied* to *very satisfied*). Educational attainment was categorised as primary school or below, junior high, senior high or university and above. Employment status (including part‐time work) was coded as yes/no.

We also assessed whether the caregivers were concerned about the vision health of the persons with IDs (yes/moderate/no). Finally, caregiver strain was measured using the four‐item Zarit Burden Interview (Bédard et al. 2001), where lower scores indicate lower levels of anticipated stress.

### 2.6 Ocular Conditions for the Persons With IDs

We included a list of ocular conditions and allowed caregivers to select one or more from the list. This question was included in the analysis to examine caregivers' perceptions of whether the persons with IDs had ocular conditions. The actual presence of these conditions was not the focus of this study. For caregivers whose family member had not attended an ophthalmologic examination, the reported conditions may reflect suspected problems, lay descriptions of symptoms or information obtained in other healthcare settings and may not represent a confirmed ophthalmologic assessment. On the other hand, for caregivers in the *user* group, reports are likely to be influenced by feedback received from eye‐care professionals. Accordingly, in all analyses, we interpret these data as indicators of caregiver perception of eye problems rather than as objective measures of ocular morbidity.

We included the following ocular conditions: refractive errors (near‐sightedness, both mild and high myopia; far‐sightedness, both mild and high hyperopia; and astigmatism, both mild and severe), strabismus, amblyopia, cataracts, presbyopia, nystagmus, ptosis, cerebral visual impairment, glaucoma, optic nerve diseases, corneal scars or degeneration, dry eye syndrome, iritis or uveitis, floaters, colour blindness, diabetic retinopathy, other retinal diseases, myopic macular degeneration, age‐related macular degeneration, unspecified macular degeneration, history of retinal detachment, trichiasis (inward‐growing eyelashes) and blindness. These conditions were selected on the basis of the literature and expert consultation (Warburg 2001a; Hsieh et al. 2025).

### 2.7 Statistical Analysis

Means and proportions were estimated using survey weights, and standard errors, confidence intervals and statistical tests accounted for the complex survey design. We were interested in whether some barriers might predominantly deter initial use of ophthalmologic services, whereas others might be more salient once services were reached. We therefore compared the prevalence of each barrier between caregivers who had ever taken the persons with IDs to an ophthalmologist (users) and those who had not (non‐users). Barriers reported significantly more often by non‐users were labelled “anticipated”, on the premise that they may prevent caregivers from making an appointment or initiating contact with services. Barriers reported significantly more often by users were labelled “experienced”, as they are more likely to reflect challenges encountered during actual ophthalmologic visits. In this study, “anticipated” and “experienced” are eye‐care‐use–based, descriptive labels derived from cross‐sectional differences between the two groups, rather than direct temporal measures of pre‐contact versus post‐contact barriers.

It should be noted that for non‐users, we interpret these barriers as expectations about taking their family member with IDs to an ophthalmologist. However, caregivers may have formed these expectations through prior encounters with healthcare services, for example, general hospital, dental or primary care appointments for the persons with IDs or for other relatives, as well as ophthalmic or optometric consultations for themselves or others. As a result, some barriers classified as “anticipated” may in fact be grounded in previous experiences within the health system. Our classification should therefore be understood as referring to the timing of barriers in relation to ophthalmologic care for the focal persons with IDs, rather than to the presence or absence of any prior healthcare exposure. This limitation is considered further in Section 4.

The prevalence of each barrier was calculated as the number and percentage of caregivers in each use group (users and non‐users and overall) who endorsed that specific barrier, with each barrier treated as a separate yes/no variable. Because multiple endorsements per caregiver were possible and some caregivers reported no barriers, the sum of counts across barriers within a column does not equal the total number of caregivers. Absolute differences in prevalence between users and non‐users and their 95% confidence intervals were calculated using the normal approximation for differences in independent proportions.

We then analysed whether caregivers' reported awareness of ocular conditions differed between users and non‐users. Differences in proportions were assessed using the design‐adjusted Rao–Scott chi‐square test (Rao and Scott 1981). Differences in means were estimated with methods that accounted for the covariance among estimates arising from the complex survey design. Variances for all analyses based on the complex survey were obtained using Taylor series linearisation.

After we had classified each barrier as anticipated or experienced on the basis of descriptive comparisons between users and non‐users, we conducted a multiple logistic regression to examine factors associated with reporting the major barrier. Among the 13 barriers, the item ‘The clinic environment is not friendly to people with intellectual disabilities’ was the only one that was reported significantly by our study population and also showed the largest absolute difference in prevalence between the two groups. Because this is the major barrier reported, we also had sufficient prevalence to support a stable survey‐weighted logistic model. In contrast, most of the experienced barriers were endorsed by fewer than 10% of caregivers, which limited the feasibility and interpretability of separate multivariable models for each of them.

To examine factors associated with reporting the primary barrier, we built a survey‐weighted logistic regression model. Candidate covariates were chosen a priori from all variables available in the survey that were conceptually related to caregivers' access to care based on prior literature (e.g., characteristics of the person with ID, caregiver sociodemographic factors, caregiving context and service use status). All selected predictors were entered simultaneously to reduce the risk of omitted‐variable bias. Continuous predictors (age of the person with ID, caregiver age, ADL, IADL and caregiver strain score) were initially specified as linear terms. We then assessed potential non‐linearity by adding quadratic terms for each of these variables and testing their contribution using design‐based Wald tests; only the quadratic term for caregiver strain improved model fit and was retained in the final model, whereas the other continuous variables were kept as linear terms. Model estimates were obtained using survey‐weighted logistic regression with Taylor‐series linearised standard errors, and overall model significance was evaluated with a design‐based Wald test of the joint association between all predictors and the outcome. All statistical analyses were performed using STATA Version 15 (StataCorp 2017).

## 3 Results

Table 1 presents the characteristics of the family caregivers and persons with IDs. Significant differences were observed between the ophthalmologic care users and non‐user groups in terms of the characteristics of the persons with IDs and their caregivers. The individuals with IDs in the user group were, on average, younger than those in the non‐user group were (mean age 31.4 vs. 37.2 years, *p* < 0.001). Several caregiver‐related characteristics also differed markedly between the groups. The caregivers in the user group were younger (*p* < 0.001), more likely to be married (71.3% vs. 57.0%, *p* < 0.001) and more often the parents of the individuals with IDs (71.3% vs. 60.0%, *p* = 0.003). They were also more likely to be employed (53.1% vs. 39.9%, *p* = 0.001) and to come from higher‐income households. For example, 15.6% of the caregivers in the user group reported an annual income of ≥ NT$800 000, whereas only 8.8% of the non‐user group reported this income level (*p* = 0.004). In addition, concerns about the vision health of the persons with IDs was significantly higher among caregivers in the user group, with 22.2% reporting ‘yes’, whereas only 8.4% did in the non‐user group (*p* < 0.001).

**TABLE 1:** Sample characteristics by ophthalmic care use.

	Total		Non‐user		User		
*n*		%		%		%	
	657		396		261		*p* ^a^
Variables for individuals with IDs							
Age (mean, SE)	34.92	0.542	37.24	0.685	31.40	0.84	< 0.001
Sex (*n*, %)							
Male	375	57.09	230	58.17	145	55.44	0.494
Female	282	42.91	166	41.83	116	44.56	
ADL (mean/SE)	81.88	0.12	81.55	0.15	82.39	0.18	0.719
IADL (mean/SE)	9.22	0.29	9.04	0.37	9.50	0.45	0.432
Has private health insurance (*n*, %)							
Yes	507	77.2	314	79.23	193	74.13	0.132
No	150	22.8	82	20.77	68	25.87	
Variables of caregivers							
Age (mean/SE)	57.3	0.50	59	0.66	54.9	0.80	< 0.001
Sex							
Male	216	32.87	138	34.88	78	29.83	0.184
Female	441	67.13	258	65.12	183	70.17	
Marital status (*n*, %)							
Married	412	62.71	226	57.04	186	71.31	< 0.001
Other	245	37.29	170	42.96	75	28.69	
Relationship with the person with IDs (*n*, %)							
Parent	424	64.51	238	60.01	186	71.34	0.003
Other	233	35.49	158	39.99	75	28.66	
Employed (*n*, %)							
Yes	297	45.17	158	39.93	139	53.12	0.001
No	360	54.83	238	60.07	122	46.88	
Household income (*n*, %)							
<NT$200 000	185	28.19	122	30.75	63	24.3	0.004
NT$200 000–NT$399 999	159	24.24	103	25.98	56	21.6	
NT$400 000–NT$599 999	167	25.49	104	26.37	63	24.15	
NT$600 000–NT$799 999	70	10.59	32	8.08	38	14.4	
≥NT$800,000	76	11.5	35	8.82	41	15.55	
Self‐reported financial status (*n*, %)							
Very dissatisfied	32	4.9	18	4.67	14	5.25	0.947
Dissatisfied	196	29.77	118	29.76	78	29.77	
Average	213	32.36	132	33.45	80	30.71	
Satisfied	193	29.39	113	28.48	80	30.76	
Very satisfied	24	3.58	14	3.63	9	3.51	
Caregiver’s educational attainment (*n*, %)							
Primary school or below	173	26.26	114	28.75	59	22.49	0.119
Junior high school	134	20.47	85	21.56	49	18.82	
Senior high school	217	33.03	126	31.72	91	35.03	
University or above	133	20.23	71	17.97	62	23.66	
Caregiver strain score (mean/SE)	11.33	0.14	11.38	0.18	11.26	0.22	0.6729
Worry about person with ID's vision health (*n*, %)							
Yes	91	13.84	33	8.36	58	22.15	< 0.001
Moderate	279	42.39	159	40.18	119	45.74	
No	288	43.77	204	51.46	84	32.11	

*Note:* Means, proportions, standard errors and statistical tests account for the complex survey design. Displayed counts are rounded survey‐weighted estimates; therefore, subgroup counts may not sum exactly to the total, and percentages may not sum exactly to 100% because of rounding.

Abbreviation: SE, standard error.

We ranked the barriers reported by family caregivers on the basis of their overall prevalence from most to least frequently cited (Table 2), where each percentage reflects the unadjusted proportion of caregivers who reported that specific barrier. The most commonly reported barrier by both groups was ‘The clinic environment is not friendly to people with IDs’. However, this concern was significantly more prevalent among non‐users (17.6%) than among users (4.5%; *p* < 0.001).

**TABLE 2:** Type of barriers reported by family caregivers.^a^

	Total		Non‐user		User		*p*	Difference^b^
	*n*	%	*n*	%	*n*	%		
	657		396		261			
The clinic environment is not friendly to people with intellectual disabilities (e.g., limited consultation space, lack of disability‐friendly features, absence of fall prevention materials or a generally unwelcoming atmosphere)	82	12.42	70	17.64	12	4.51	< 0.001	13.13
Health appointments or examinations may require communication support and accommodations	43	6.52	17	4.25	26	9.95	0.004	−5.7
Medical instruments and equipment are unsuitable for the person with intellectual disabilities (e.g., lack of portable devices for refraction or intraocular pressure measurement)	38	5.75	17	4.25	21	8.04	0.043	−3.79
There is a lack of assessment tools adapted for people with intellectual disabilities (e.g., visual acuity charts and colour blindness tests)	33	4.95	14	3.53	19	7.09	0.043	−3.56
Health‐care personnel lack knowledge and skills related to intellectual disabilities (e.g., difficulties in communicating with individuals with intellectual disabilities)	30	4.55	10	2.55	20	7.59	0.003	−5.04
There is no time to take the person with intellectual disabilities to the appointment	29	4.37	14	3.44	15	5.77	0.155	−2.33
The medical facility is too far away or transportation is inconvenient	28	4.36	11	2.85	17	6.65	0.018	−3.8
Healthcare personnel are impatient or unfriendly towards the person with intellectual disabilities	28	4.27	15	3.7	13	5.14	0.374	−1.44
The financial burden is too high or treatment is too expensive	19	2.82	4	1.01	15	5.56	0.001	−4.55
Other patients or their family members in the clinic are unfriendly	16	2.57	6	1.63	10	4	0.054	−2.37
I believe that the treatment would be ineffective	13	1.99	5	1.21	8	3.18	0.082	−1.97
I am unable to secure an appointment with the desired physician	5	0.83	0	0	5	2.09	0.006	−2.09
I do not understand the different treatment options, making decision‐making difficult	5	0.7	4	0.93	1	0.34	0.351	0.59

^a^ Each row shows the number (percentage) of caregivers who reported that barrier. Caregivers could select more than one barrier or none at all; therefore, the sums of counts and percentages across barriers within a column do not equal the total sample size and do not sum to 100%.

^b^ Expressed as percentage points.

Several other barriers were significantly more likely to be reported by caregivers who had already taken the persons with IDs to an eye clinic. These included challenges engaging during examinations (9.95% among users vs. 4.25% among non‐users, *p* = 0.004), unsuitable equipment (8.04% vs. 4.25%, *p* = 0.043), a lack of assessment tools adapted to the needs of people with IDs (7.09% vs. 3.53%, *p* = 0.043), a lack of provider knowledge or skills related to IDs (7.59% vs. 2.55%, *p* = 0.003), inconvenient transportation (6.65% vs. 2.85%, *p* = 0.018), high cost (5.56% vs. 1.01%, *p* = 0.001) and difficulty obtaining an appointment (2.09% vs. 0%, *p* = 0.006).

Although several experienced barriers were identified on the basis of our definitions, the overall proportion of caregivers reporting each of these barriers was relatively low (all below 10%). For most barriers, absolute differences in prevalence between users and non‐users were modest, typically in the range of 2–6 percentage points. For example, caregivers of users were 5.7 percentage points (95% CI 1.5–9.8) more likely than non‐users to report that the persons with IDs had difficulty participating during visits and 4.6 percentage points (95% CI 1.7–7.7) more likely to report financial burden as a barrier.

Table 3 summarises caregiver‐reported ocular conditions for the persons with IDs according to ophthalmologic service use status. Overall, caregivers in the user group were more likely than those in the non‐user group to report that the persons with IDs had one or more eye conditions. Because these data are based on caregiver report without clinical verification, they should be interpreted as differences in suspected problems rather than as definitive differences in ocular morbidity.

**TABLE 3:** Caregiver‐reported ocular conditions of the person with intellectual disabilities, by ophthalmologic care use status.^a^

	Total		Non‐user		User		*p*
	*n* = 657		*n* = 396		*n* = 261		
	*n*	%	*n*	%	*n*	%	
Myopia (< 500°)	80	12.18	38	9.60	42	16.09	0.014
High myopia (>= 500°)	26	3.96	13	3.28	13	4.98	0.306
Hyperopia (< 300°)	6	0.91	0	0	6	2.30	0.004
High hyperopia (>= 300°)	1	0.15	0	0	1	0.38	0.393
Astigmatism (< 250°)	33	5.02	14	3.54	19	7.28	0.043
High astigmatism (> 250°)	3	0.46	1	0.25	2	0.77	0.565
Colour blindness	1	0.15	0	0	1	0.38	0.393
Strabismus	31	4.72	7	1.77	24	9.20	< 0.001
Amblyopia	31	4.72	8	2.02	23	8.81	< 0.001
Cataract	17	2.59	6	1.52	11	4.21	0.042
Glaucoma	1	0.15	0	0	1	0.38	0.393
Presbyopia	21	3.20	16	4.04	5	1.92	0.175
Age‐related macular degeneration	1	0.15	1	0.25	0	0	1.000
Diabetic retinopathy	2	0.30	1	0.25	1	0.38	1.000
Previous retinal detachment	3	0.46	1	0.25	2	0.77	0.565
Optic neuropathy	2	0.30	1	0.25	1	0.38	1.000
Nystagmus	4	0.61	2	0.51	2	0.77	0.648
Dry eye syndrome	2	0.30	1	0.25	1	0.38	1.000
Iritis or uveitis	1	0.15	0	0	1	0.38	0.393
Cerebral visual impairment	3	0.46	0	0	3	1.15	0.060
Trichiasis	1	0.15	0	0	1	0.38	0.393
Blindness	10	1.52	8	2.02	2	0.77	0.330

^a^ Ocular conditions are reported by caregivers and were not clinically verified in this study; therefore, they represent perceptions rather than objective prevalence.

Table 4 presents adjusted average marginal effects from the logistic regression model for reporting the barrier of an unfriendly clinic environment. After controlling for all covariates, non‐users were more likely than users to report this barrier, with a 9.7‐percentage‐point difference in predicted probability (*p* < 0.001). Male caregivers were 5.9 percentage points more likely than female caregivers to report this barrier (*p* = 0.023). Caregivers who were not worried about the vision health of the persons with IDs were 18.3 percentage points more likely to endorse the barrier than those who reported being worried (dy/dx = 0.183, 95% CI 0.126–0.241, *p* < 0.001). Higher caregiver strain was also associated with barrier perception; each 1‐point increase in the strain score was associated with a 1.7‐percentage‐point increase in the probability of reporting the barrier (dy/dx = 0.017, 95% CI 0.010–0.024, *p* < 0.001). In addition, caregivers who reported that the persons with IDs had two or more ocular conditions were 18.3 percentage points more likely to endorse the barrier than those who reported no ocular condition (dy/dx = 0.183, 95% CI 0.074–0.291, *p* = 0.001). Other characteristics, including age, ADL and IADL scores, household income and educational attainment, were not significantly associated with reporting this barrier after adjustment.

**TABLE 4:** Marginal effect of explanatory variables on reporting anticipated barriers.^a^

	Unadjusted				Adjusted			
	dy/dx	95% confidence interval		*p*	dy/dx	95% confidence interval		*p*
Variables of persons with IDs								
Age	0.0006	−0.0012	0.0024	0.4920	0.0002	−0.0017	0.0020	0.8720
Sex								
Male (reference)								
Female	−0.0237	−0.0745	0.0272	0.3610	−0.0123	−0.0579	0.0333	0.5960
ADL	0.0002	−0.0008	0.0012	0.7320	0.0003	−0.0007	0.0013	0.5160
IADL	−0.0026	−0.0061	0.0009	0.1390	−0.0017	−0.0054	0.0021	0.3880
Has private health insurance								
No (reference)								
Yes	−0.0385	−0.0905	0.0134	0.1460	−0.0413	−0.0904	0.0078	0.0990
Caregiver's variables								
Age	0.0013	−0.0005	0.0031	0.1590	0.000089	−0.00215	0.002328	0.938
Sex								
Male (reference)								
Female	−0.0402	−0.0965	0.0161	0.1610	−0.05853	−0.10913	−0.00793	0.023
Marital status								
Others (reference)								
Married	−0.0031	−0.0553	0.0492	0.9090	0.0010	−0.0467	0.0488	0.9670
Relationship with persons with IDs								
Other (reference)								
Parents	0.0303	−0.0209	0.0814	0.2460	0.0002	−0.0572	0.0576	0.9940
Household income								
< NT200 000 (reference)								
NT 200000 ~ 399 999	0.0882	0.0162	0.1601	0.0160	0.0300	−0.0390	0.0990	0.3930
NT 400 000–599 999	0.0481	−0.0176	0.1137	0.1510	−0.0313	−0.0975	0.0350	0.3550
NT 600 000–799 999	0.0548	−0.0364	0.1460	0.2390	−0.0162	−0.0983	0.0659	0.6990
> = NT 800 000	0.0024	−0.0722	0.0770	0.9500	−0.0545	−0.1470	0.0380	0.2470
Self‐reported financial status								
Very dissatisfied (reference)								
Dissatisfied	0.0057	−0.1015	0.1130	0.9170	−0.0274	−0.1413	0.0866	0.6380
Average	0.0447	−0.0646	0.1539	0.4220	0.0215	−0.0932	0.1362	0.7130
Satisfied	0.1016	−0.0121	0.2153	0.0800	0.1175	−0.0004	0.2354	0.0510
Very satisfied	0.0902	−0.0904	0.2709	0.3270	0.1286	−0.0732	0.3305	0.2110
Caregiver's highest education								
Primary school or below (reference)								
Junior high school	0.0040	−0.0744	0.0825	0.9200	−0.0303	−0.1060	0.0454	0.4320
Senior high school	−0.0079	−0.0762	0.0604	0.8210	−0.0206	−0.0862	0.0451	0.5380
University or above	−0.0526	−0.1211	0.0160	0.1320	−0.0634	−0.1309	0.0041	0.0660
Currently employed								
No (reference)								
Yes	0.0067	−0.0441	0.0574	0.7960	0.0314	−0.0207	0.0834	0.2370
Worry about person with ID's vision health								
Yes (reference)								
Moderate	0.0013	−0.0379	0.0406	0.9480	−0.0005	−0.0432	0.0422	0.9810
No	0.2153	0.1556	0.2749	0.0000	0.1831	0.1257	0.2405	< 0.0001
Has taken person with IDs for eye exam								
No (reference)								
Yes	−0.1312	−0.1763	−0.0861	0.0000	−0.0966	−0.1399	−0.0534	<0.0001
Caregiver strain score	0.0178	0.0111	0.0245	0.0000	0.0171	0.0097	0.0244	<0.0001
Number of ocular conditions reported								
0 (reference)								
1	−0.0388	−0.0933	0.0157	0.1630	0.0116	−0.0408	0.0640	0.6650
> =2	0.0431	−0.0658	0.1520	0.4370	0.1826	0.0740	0.2912	0.0010
F_27,629_ = 2.93								

^a^ The anticipated barrier is ‘The clinic environment is not friendly to persons with intellectual disabilities (e.g., limited consultation space, lack of disability‐friendly features, absence of fall prevention materials or a generally unwelcoming atmosphere)’.

## 4 Discussion

In the present study, we compared barriers to ophthalmologic care reported by family caregivers of users and non‐users and interpreted these differences as anticipated or experienced barriers. The key findings were as follows: (1) The perception that the clinic environment is unfriendly to people with IDs was the only barrier significantly more common among non‐users than among users and was therefore interpreted as an ‘anticipated’ barrier. Several additional barriers were identified among caregivers who had used ophthalmologic services. These patterns are consistent with our first hypothesis that people with IDs encounter multiple barriers to ophthalmologic care, and with our second hypothesis that barriers reported by non‐users may reflect expectations about the care setting, whereas barriers reported by users may reflect challenges arising from actual service engagement. (2) For all ‘experienced’ barriers, the proportion of caregivers reporting each individual barrier remained relatively low. These barriers were reported by fewer than 10% of caregivers in either eye‐care‐use group, and for the experienced barriers, the absolute differences in prevalence between users and non‐users were generally modest. Thus, with the exception of the anticipated barrier of an unfriendly clinic environment, our results highlight barriers that affect a minority of families rather than obstacles that are universally experienced. (3) In addition to actual use of ophthalmologic care, the factors associated with a higher likelihood of reporting an anticipated barrier included being a male caregiver, not expressing concern about the vision health of the person with IDs, having a higher caregiver strain score and perceiving that the person with IDs had two or more ocular conditions. Caregivers who had taken the person with IDs for vision care were more likely to report that the individual had ocular conditions, consistent with Hypothesis 3. A key strength of this study is its use of a large and regionally representative sample from both rural and urban areas of Taiwan, which is rare in research on ophthalmologic care for people with IDs.

Dedicated ophthalmology clinics play a critical role in evaluating and treating people with IDs because vision care for this population often involves complex procedures and requires considerable time and accommodation (Van Isterdael et al. 2006). Our study contributes to the literature by indicating that a high proportion of non‐users anticipated that the clinic environment would be unfriendly to people with IDs, despite never having taken their family member to an ophthalmology clinic. Caregivers who have not taken their family member with IDs to an ophthalmologic clinic may hold assumptions or perceive this as a major barrier that could discourage them from seeking care.

One possible explanation for this is that caregivers may consider their previous negative experiences with non‐ophthalmologic healthcare settings in making such judgements. Gibbs et al. (2008) reported that previous negative interactions with hospital environments affected how people with IDs felt during their subsequent visits. In the current study, friendly clinical settings for people with IDs may be lacking in general care, leading caregivers to assume that similar shortcomings will be present in ophthalmology clinics. For some caregivers, their perceptions about the clinic environment may be particularly salient in decisions about whether to seek ophthalmologic care. These patterns may mean that what we have termed ‘anticipated’ barriers are likely to be shaped, at least in part, by prior experiences in other healthcare environments and therefore represent expectations about ophthalmologic care rather than barriers that are entirely hypothetical.

The respondents in the present study were family caregivers rather than people with IDs themselves. Thus, their perceptions of barriers may not fully align with those of the individuals they care for. For example, studies focusing on people with IDs' perspectives have found that they may describe fear linked to uncertainty about what will happen during care (Gibbs et al. 2008; Chou et al. 2025). However, because caregivers are generally more informed about procedural steps, they may not have this fear, which could explain the relatively low percentage of reported barriers among caregivers in the user group. Although some individuals with IDs did not use oral communication, many demonstrated a clear understanding of medical care and procedures (Gibbs et al. 2008). This is consistent with our finding that caregivers who had taken the person with IDs to an ophthalmologist reported fewer communication‐related barriers than did those who had not. In addition, other patients in the clinical room may not be as unfriendly as anticipated (Gibbs et al. 2008).

It is possible that some barriers are more likely to become apparent through direct experience with care. In other words, although environmental concerns may deter initial access, challenges that require participation supports during visits, the availability of appropriate tools and provider preparedness are more commonly reported by caregivers who have already accessed services.

Among the non‐users, only a small proportion reported barriers other than an unfriendly clinic environment. These patterns may indicate that the expectation of an inhospitable clinical setting may be so strong among caregivers that it alone is sufficient to deter them from seeking ophthalmologic care. In practical terms, even when support such as transportation, financial assistance or provider training is available, caregivers may avoid scheduling an appointment because of concerns about the clinical environment. These concerns may include cramped spaces, inaccessible equipment or uncertainty about whether healthcare staff and ophthalmologists will be friendly and accommodating (Chou et al. 2025). Caregivers may also worry about negative reactions from other patients in shared waiting rooms (Gibbs et al. 2008), even if such reactions are not always as common or as severe as anticipated.

We observed that caregivers in the user group were significantly more likely to report that the person with IDs had specific ocular conditions than caregivers in the non‐user group. Given that our measure relies on caregiver report and many items correspond to clinical assessment, this pattern is most plausibly interpreted as reflecting greater awareness generated through contact with eye‐care providers, rather than a true difference in underlying ocular morbidity between the groups. Previous studies that used standardised ophthalmologic examinations have shown that many vision problems among people with IDs remain undetected or untreated until proactive screening is undertaken (Van Splunder et al. 2006; Warburg 2001b; Donaldson et al. 2019). Our data cannot directly quantify such unmet clinical need because we did not obtain independent clinical verification of ocular status. Instead, it is possible that caregivers who have never engaged with ophthalmologic services may have limited information about possible eye conditions in their family member and that anticipated environmental barriers that deter service use may indirectly contribute to this lack of awareness.

Ophthalmology clinics specifically designed to accommodate people with IDs may not always be available (Hou et al. 2025). Our findings may indicate that visibly improving clinic environments could be a particularly relevant starting point. Rather than attempting to address all possible barriers simultaneously, policies could prioritise interventions that enhance the perceived friendliness and accessibility of clinic spaces. Simple design changes, such as expanding the consultation space, incorporating disability‐friendly physical layouts, adding safety features to prevent falls and creating a warm, welcoming atmosphere, can pre‐emptively counteract negative assumptions that deter care‐seeking. Community‐based initiatives and outreach programmes also play a critical role in supporting access to vision care for people with IDs (Ellison 2013). Disability‐affirming care experiences may help counteract the gatekeeper effect of anticipated environmental barriers, particularly for families who are most concerned about the clinic environment.

This study has several limitations that should be considered. First, our distinction between anticipated and experienced barriers is based on differences in reporting between non‐users and users and does not fully disentangle expectations from prior experiences of care. Our categories should therefore be interpreted as indicating whether barriers were reported before or after an ophthalmology visit for the focal person with IDs, rather than as a strict contrast between expectations and experiences in an absolute sense. Future research could address this limitation by collecting detailed information on caregivers' prior healthcare use and by asking respondents to indicate, for each barrier, whether it reflects personal experiences or anticipated difficulties.

Second, the cross‐sectional design of the study precludes causal inference. Although we observed that anticipated barriers are associated with non‐use of vision care, we cannot determine whether these barriers directly cause caregivers to avoid seeking care or instead reflect underlying factors, such as prior healthcare experiences and structural disadvantage.

## 5 Conclusion

Our study highlights differences between barriers reported by caregivers of non‐users and users of ophthalmologic care for people with IDs, which we interpreted as anticipated and experienced barriers. The higher reporting of an unfriendly clinic environment among non‐users suggests that this perception may deter first contact with ophthalmologic care services. Policy efforts should therefore prioritise improving clinical environments, both in terms of physical space and appointment procedures, to address this potential barrier.

## Funding

This study was supported by the National Science and Technology Council, Taiwan (Grant Numbers NSCT 114‐2314‐B‐A49‐032‐MY3, NSCT 112‐2314‐B‐A49‐052‐MY3 and NSTC 114‐2423‐H‐468‐001).

## Conflicts of Interest

The authors declare no conflicts of interest.

## Data Availability

The data that support the findings of this study are not publicly available due to privacy and ethical restrictions. Access to the data may be granted upon reasonable request to the corresponding author, provided that approval is obtained from the institutional review board/ethics committee responsible for approving the original study.
